# Pre-procedural proton pump inhibition is associated with fewer peri-oesophageal lesions after cryoballoon pulmonary vein isolation

**DOI:** 10.1038/s41598-021-83928-0

**Published:** 2021-02-25

**Authors:** F. Cordes, C. Ellermann, D. G. Dechering, G. Frommeyer, S. Kochhäuser, P. S. Lange, C. Pott, F. Lenze, I. Kabar, H. Schmidt, H. Ullerich, L. Eckardt

**Affiliations:** 1 Department of Medicine B, Gastroenterology and Hepatology, University Hospital Muenster, Muenster, Germany; 2 Department of Cardiology II (Electrophysiology), University Hospital Muenster, Albert-Schweitzer Campus 1, 48149 Münster, Germany; 3 Department of Cardiology, Schuechtermann-Klinik, Bad Rothenfelde, Germany

**Keywords:** Risk factors, Digestive signs and symptoms, Interventional cardiology, Oesophagogastroscopy

## Abstract

Pulmonary vein isolation (PVI) using cryoenergy is safe and efficient for treatment of atrial fibrillation (AF). Pre-existing upper gastrointestinal (GI) pathologies have been shown to increase the risk for AF. Therefore, this study aimed at assessing incidental pathologies of the upper GI tract in patients scheduled for PVI and to analyse the impact of patients’ characteristics on PVI safety outcome. In 71 AF patients, who participated in the MADE-PVI trial, oesophagogastroduodenoscopy and endosonography were prospectively performed directly before and the day after PVI to assess pre-existing upper GI pathologies and post-interventional occurrence of PVI-associated lesions. Subgroup analysis of the MADE-PVI trial identified clinically relevant incidental findings in 53 patients (74.6%) with age > 50 years being a significant risk factor. Pre-existing reflux oesophagitis increased risk for PVI-associated mediastinal oedema, while patients already treated with proton pump inhibitors (PPI) had significantly fewer mediastinal oedema. Our results suggest that AF patients with pre-existing reflux oesophagitis are at higher risk for PVI-associated mediastinal lesions, which is decreased in patients with constant PPI-treatment prior to PVI. Since PVI-associated mediastinal lesions are regarded as surrogate parameter for an increased risk of the fatal complication of an oesophago-atrial fistula, our findings hint at a beneficial effect of pre-interventional prophylactic PPI-treatment to reduce risk for PVI-associated complications.

German Clinical Trials Register (DRKS00016006; date of registration: 17/12/2018).

## Introduction

Atrial fibrillation (AF) is the most common heart rhythm disorder with a significant association with morbidity and mortality^1^. Patients with symptomatic AF are increasingly frequently treated by catheter ablation. Pulmonary vein isolation (PVI) is regarded as the cornerstone in interventional treatment and can be achieved by employing different energy sources such as radiofrequency or cryoenergy^2^. Independent of the energy source, one of the most feared complications of PVI is damage of the oesophagus, which can ultimately lead to the highly fatal development of an atrio-oesphageal fistula^3,4^.

Much effort has been expended in order to reduce damage of the oesophagus due to catheter ablation of AF. However, predisposing, or even more importantly, protective factors for the development of atrio-oesophageal fistula are hard to identify due to the fortunately low prevalence of atrio-oesophageal fistula. Determination of these factors is even more complicated by the fact that the pathophysiology of atrio-oesophageal fistula is not well understood. However, since formation of an atrio-oesophageal fistula occurs usually weeks after the ablation procedure, a likely explanation includes injury of peri-oesophageal vessels with subsequent ischemic lesions of the oesophagus^5^. Therefore, mediastinal and oesophageal lesions might be regarded as precursor lesions of an atrio-oesophageal fistula.

In the recently published MADE-PVI (Mediastino-oesophageal Alterations Detected by Endosonography after PVI) study^6^, the ablation protocol employing 2nd generation cryoballoon had a distinct impact on oesophageal and mediastinal lesions. A time-to-isolation (TTI)-guided ablation protocol (TTI + 120 s) was associated with significantly less mediastinal and oesophageal lesions and might therefore reduce the risk for oesophageal damage compared to a fixed-time freeze application (2 × 180 s). In addition to the ablation protocol, low body weight, old age, and high CHA_2_DS_2_-VASc-scores seem to be associated with an increased risk for atrio-oesophageal fistula^7^.

Many comorbidities such as obesity, obstructive sleep apnoea or diabetes mellitus favour the development not only of AF but also of morbidities of the upper gastrointestinal (GI) tract^8^, indicating that AF patients are at increased risk for upper GI disorders. Therefore, aim of the present study was to assess the incidence of upper GI pathologies in patients scheduled for cryoballoon ablation and to identify factors that predispose to the post-procedural development of mediastinal and oesophageal lesions.

## Methods

### The MADE-PVI trial

The design and results of the MADE-PVI study have been reported in detail^6^. In brief, MADE-PVI was a prospective, controlled single-centre trial and conducted to investigate whether the ablation strategy employing 2nd generation cryoballoon had an impact on the occurrence of oesophageal and mediastinal lesions. The study was approved a priori by the ethics committee of the Westfälische Wilhelms-Universität Münster (No: 2017-325-f-S), has been performed in accordance with the 1975 Declaration of Helsinki and its later amendments and is registered at the German Clinical Trials Register (DRKS00016006; date of registration: 17/12/2018). All persons gave their written informed consent to participate prior to their inclusion in the study.

Symptomatic patients scheduled for PVI were prospectively enrolled after giving informed consent. Eligible patients were between 18 and 80 years old and did not meet any of the following exclusion criteria: previous left atrial ablation procedure or surgery, functional class IV of the New York Heart Association classification, left ventricular ejection fraction ≤ 30%, severe chronic obstructive pulmonary disease, presence of a left atrial, or ventricular thrombus, pregnancy, contraindications to oral anticoagulation or known severe oesophageal disorders including history of oesophageal cancer, achalasia, eosinophil oesophagitis, oesophageal stenosis as well as of oesophageal operations.

In all patients, cryoballoon PVI was performed as described elsewhere in detail^6^. Two different ablation strategies were pursued: either a conventional protocol which consisted of 2 × 180 s freezes or a TTI-guided ablation protocol were employed. For the TTI-guided approach, freeze time was determined as TTI plus 120 s or, in case no real-time pulmonary vein potentials could be recorded, a single 180 s freeze was delivered.

Each patient underwent endoscopic examination prior and the day after PVI. Oesophagogastroduodenoscopy (EGD) and endoscopic ultrasound (EUS) were employed in order to identify ablation-associated oesophageal and mediastinal changes. Furthermore, pre-procedural EGD and EUS enabled determination of pre-existing upper GI pathologies.

### Patient characteristics

As previously in part described^6^, 71 patients with AF planned for PVI were included after obtaining informed consent. Patients had no previous AF ablation. Median age of patients was 60 ± 10 years, average BMI was 27.4 ± 3.6 kg/m^2^, 75% (n = 53) of patients were male. Most frequent comorbidities included hypertension (n = 37, 52%), diabetes mellitus (n = 5, 7%) and ischemic/non-ischemic cardiomyopathy (n = 11, 15.5%). Patients’ characteristics of the MADE-PVI cohort are described in Supplemental^6^ Table 1. Subanalyses of the MADE-PVI cohort included assessment of incidental findings in patients aged older versus younger than 50 years (Supplemental Table 2) as well as of obese compared to non-obese patients with a BMI of 30 kg/m^2^ as a cut-off (Supplemental Table 3).

**Table 1:** Incidental findings of the MADE-PVI cohort prior to PVI.

Incidental findings	Patients
Erosive oesophageal reflux disease [n (%)]	
Mild (A—LA-classification)	16 (22.5)
Moderate (B—LA-classification)	5 (7.0)
Severe (C/D—LA-classification)	1 (1.4)
Barrett´s oesophagus [n (%)]	14 (19.7)
Mycosal oesophagitis [n (%)]	4 (5.6)
Hiatal hernia [n (%)]	20 (27.7)
Gastritis [n (%)]	
Mild (erythematous gastritis)	7 (9.9)
Moderate (striped gastritis)	29 (40.9)
Severe (erosive/ulcerative gastritis)	6 (8.5)
Ulcer [n (%)]	5 (7.0)
Polypoid lesions [n (%)]	
Body gland cysts	9 (12.7)
Submucosal lesion	5 (7.0)
Mucosal lesions	1 (1.4)
Malignoma [n (%)]	1 (1.4)

IQR, inter-quartile-range; n, number; PVI, pulmonary vein isolation.

Additionally, treatment with proton-pump inhibitors (PPI) was analysed in the MADE-PVI cohort. In detail, patients with PPI treatment prior to PVI (classified as PPI pre-treatment) were identified. PPI pre-treatment was defined as constant daily PPI therapy, which started at least 4 weeks prior to PVI. PPI pre-treatment was found in 14 patients including medication with pantoprazole (n = 12) and omeprazole (n = 2). The doses ranged from 20 mg (n = 2) to 40 mg (n = 10) in pantoprazole treated patients and from 20 mg (n = 1) to 40 mg (n = 1) in patients with omeprazole treatment. Supplemental Table 4 describes demographics and characteristics of patients with PPI pre-treatment.

### Statistics

Data of patients were prospectively assessed prior and post-PVI. Data analysis was performed with the IBM SPSS®, V.24 (IBM Corporation, Somers, NY, USA) software. Analysis of categorical data was performed by using the Chi-square test and Fisher’s exact test, respectively, when appropriate. Non-Gaussian continuous data were analysed using the Mann–Whitney U test. Univariable and multivariable binary logistic regression analyses were used to identify risk factors for mediastinal oedemas. *P*-values ≤ 0.05 were considered as statistically significant.

## Results

### Incidental endoscopic findings in AF patients

**Figure 1:**
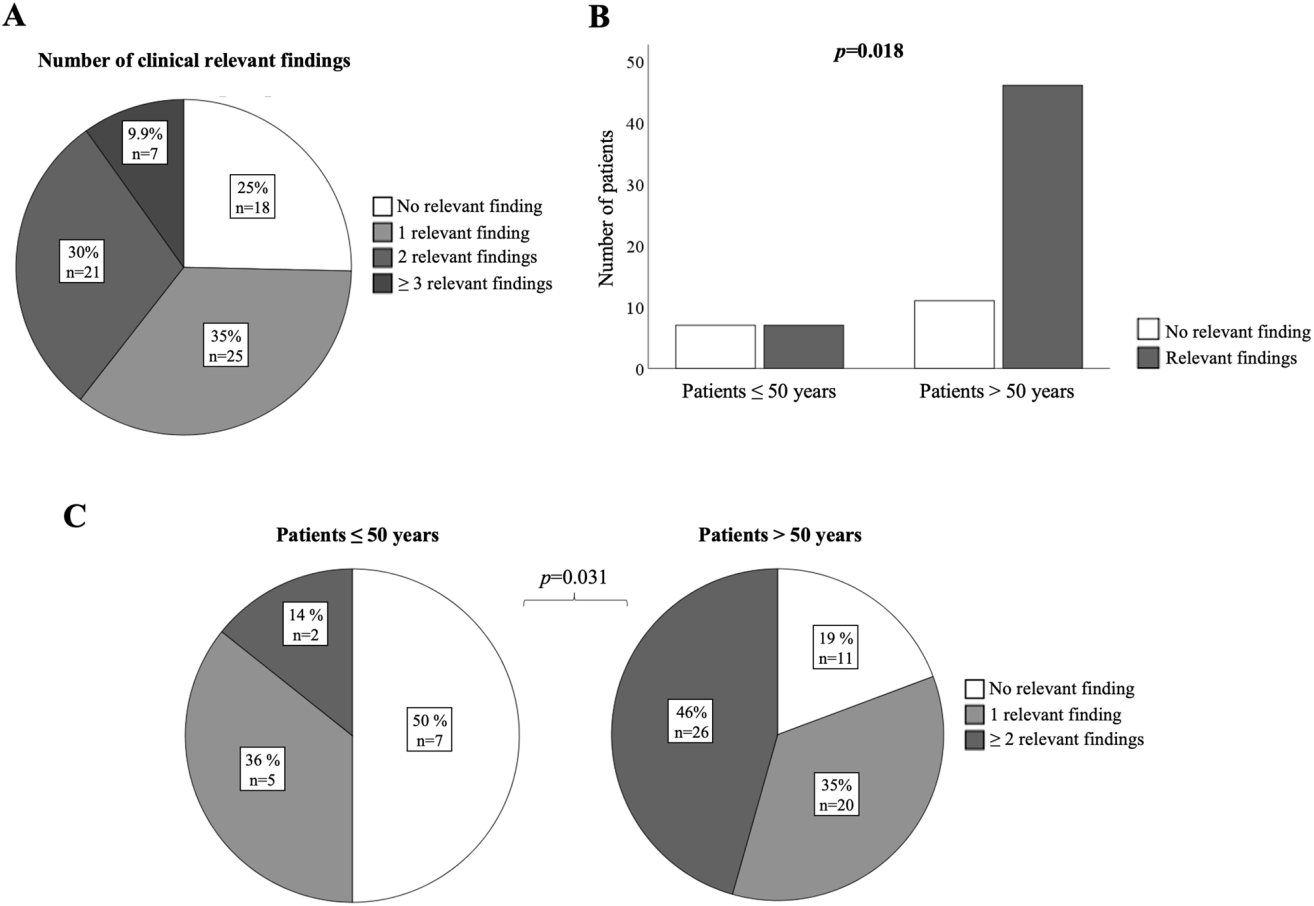
Incidental findings of the MADE-PVI cohort prior to PVI. (**A**) Clinically relevant incidental findings prior to PVI occurred in 53 out of 71 patients (74.6%). Out of these patients, 25 patients suffered from one incidental finding, while 21 and 7 patients suffered from 2 or more clinically relevant incidental findings, respectively. Shown is the proportion / number of patients with clinically relevant incidental findings. (**B**) Clinically relevant incidental findings were detected significantly more frequently in patients aged older than 50 years compared to younger patients (*p* = 0.018). (**C**) Patients aged older than 50 years had a distinctly higher risk for more than one pathology of the upper gastrointestinal tract compared to younger patients (*p* = 0.031). Shown is the number (**B**) and proportion (**C**) of patients with clinically relevant incidental findings.

Incidental endoscopic findings with clinical relevance were defined as findings with diagnostic or therapeutic relevance and were observed in 74.6% (n = 53) of patients, including reflux oesophagitis (n = 22, 31.0%), Barrett´s oesophagus (n = 14, 19.7%), oesophageal candida mycosis (n = 4, 5.6%), moderate to severe gastritis (n = 35, 49.3%) excluding mild gastritis (n = 7, 9.9%), gastric ulcer (n = 5, 7.0%), malignoma (n = 1, 1.4%) and polypoid mucosal or submucosal lesions (n = 15, 21.1%, of which were nine body gland cysts (12.7%). Detailed analyses of all incidental findings are described in Table 1. More detailed, 35.2% of the patients (n = 25) had one incidental clinically relevant finding, while 30.0% (n = 21) and 9.9% (n = 7) had two and more incidental findings with clinical relevance, respectively (Fig. 1A).

### Impact of age, BMI and gender on incidental endoscopic findings

Next, we analysed the impact of age with a cut-off of 50 years on upper GI pathologies in our AF cohort. Indeed, overall clinically relevant incident endoscopic findings were detected more frequently (*p* = 0.018) and at a higher cumulative number (*p* = 0.031) in patients older than 50 years (Fig. 1B,C). In detail, moderate to severe gastritis (*p* = 0.02) was found more frequently in patients > 50 years compared to younger patients (Supplemental Table 2). Of note, none of the patients younger than 50 years had deep gastral erosions or ulcer compared to 16% (n = 9) of patients aged older than 50 years (p = 0.112). Furthermore, Barrett´s oesophagus was only detected in 1/14 (7.1%) patients younger than 50 years, while 13/57 (22.8%) patients older than 50 years had an incidental finding of Barrett´s oesophagus (*p* = 0.187). Nevertheless, these findings were not statistically significant, possibly due to the small cohort. Occurrence of mild to severe reflux oesophagitis according to the Los Angeles (LA) classification^9^, oesophageal mycosis or mucosal and submucosal lesions were not significantly different in AF patients older or younger than 50 years. Of note, gender, BMI, comorbidities and medication did not differ between both cohorts (Supplemental Table 2).

Next, we assessed the impact of the body mass index (BMI) on clinically relevant endoscopic findings. While there was no significant difference concerning overall clinically relevant endoscopic findings in patients with a BMI < 30 kg/m^2^ compared to obese patients (> 30 kg/m^2^, Supplemental Fig. 1A), analysis of the occurrence of erosive oesophageal reflux revealed that in obese patients moderate to severe reflux oesophagitis (B-D according to LA classification) was detected significantly more frequently compared to the patient cohort with a BMI < 30 kg/m^2^ (*p* = 0.044, Supplemental Fig. 1B, Supplemental Table 3). Gender had no significant impact on endoscopic findings in AF patients.

### Impact of pre-existing upper GI pathologies and protone pump inhibitor pre-treatment on PVI safety outcome

**Figure 2:**
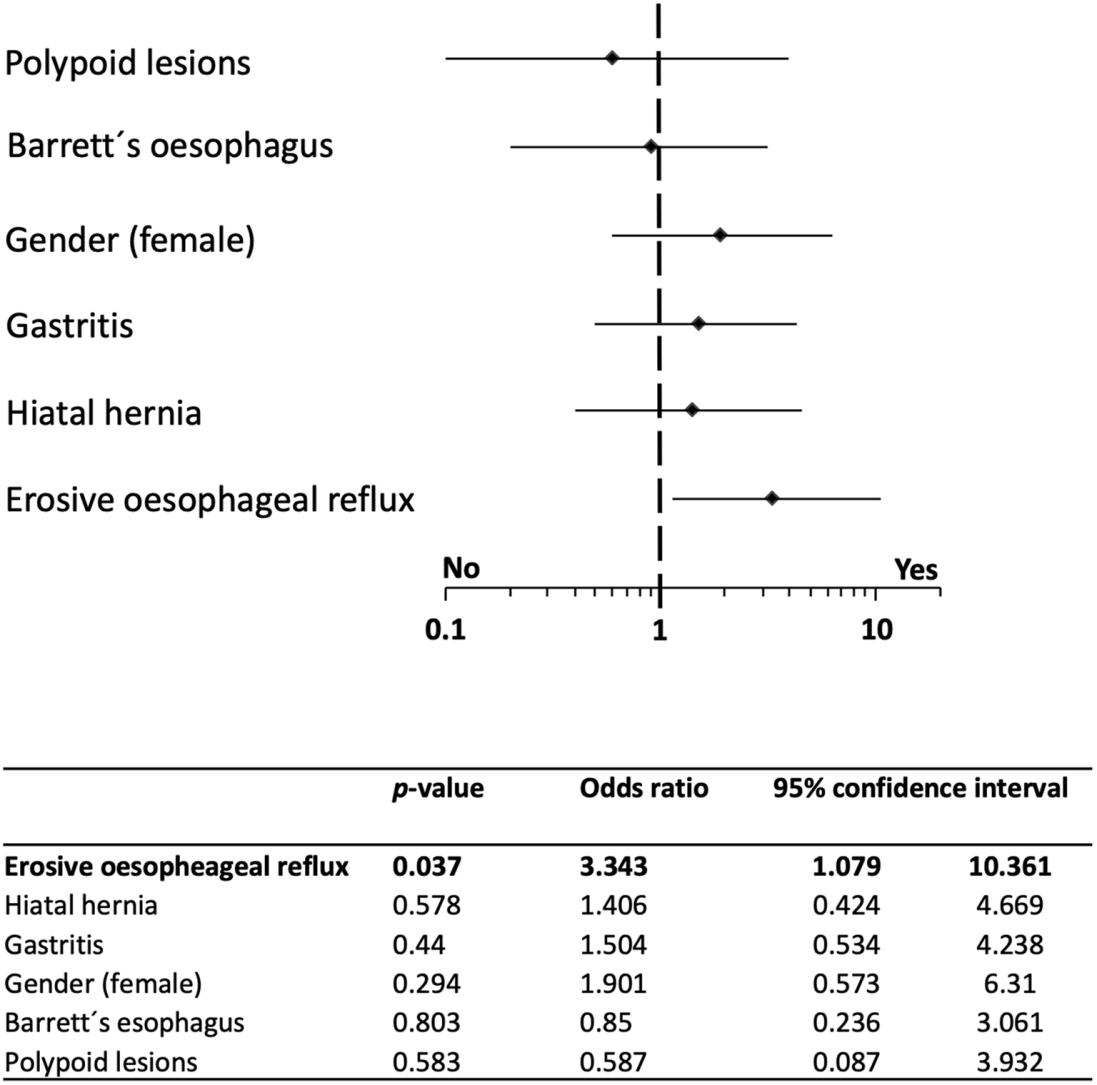
Analysis of pre-existing upper gastrointestinal pathologies as risk factors for PVI-associated mediastinal oedema. Multinomial logistic regression to calculate odds ratios with 95% confidence intervals identified mild to severe erosive oesophageal reflux disease as risk factors for post-ablation mediastinal oedema. PVI, pulmonary vein isolation.

**Figure 3:**
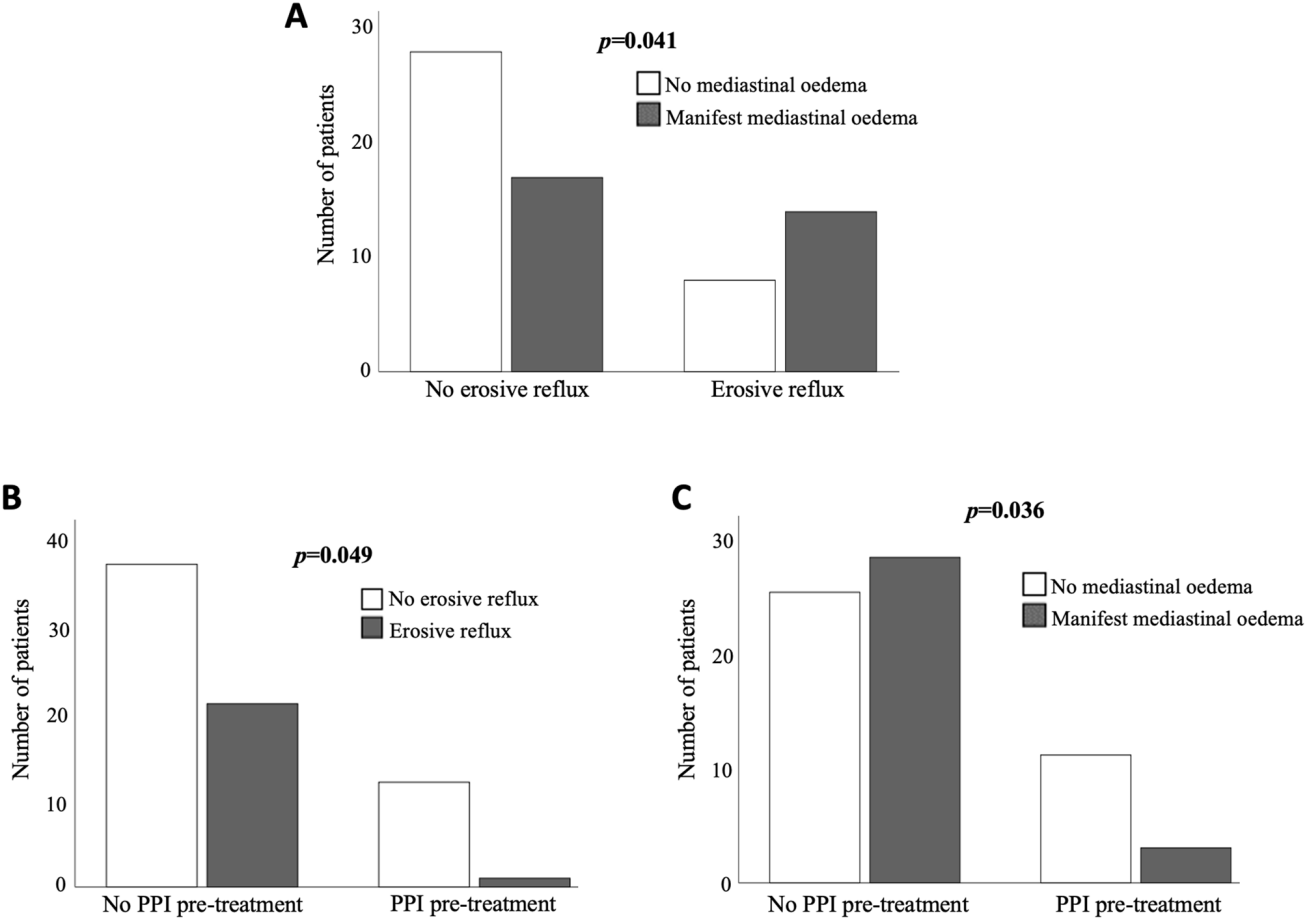
Impact of oesophageal reflux disease and prior PPI-treatment on PVI safety outcome. (**A**) PVI-associated mediastinal oedema were significantly more frequently detected in patients with pre-existing erosive oesophageal reflux disease compared to non-reflux patients (*p* = 0.041). Shown is the number of patients with or without mediastinal oedema after PVI. (**B**) Patients with constant PPI medication prior to PVI significantly suffered less frequently from erosive oesophageal reflux disease, assessed directly prior to PVI by EDG, compared to patients without prior constant PPI intake (*p* = 0.049). Shown is the number of patients with or without erosive oesophageal reflux prior to PVI. (**C**) In patients with constant PPI-treatment prior to PVI, PVI-associated mediastinal oedema were significantly less frequently observed by post-interventional endosonography (*p* = 0.036). Shown is the number of patients with or without mediastinal oedema after PVI. EGD, oesophagogastroduodenoscopy; PPI, proton pump inhibitor; PVI, pulmonary vein isolation.

Finally, we analysed the impact of asymptomatic pre-existing pathologies of the upper GI tract on occurrence of PVI-associated post-interventional oesophago-mediastinal changes. While pre-existing gastritis, gastric/duodenal ulcer disease or Barrett´s oesophagus without acute inflammation had no influence, we could detect a significantly increased risk for mediastinal oedema in patients with pre-existing reflux oesophagitis compared to non-oesophagitis AF patients in a binary regression analysis (OR 3.3; 95% CI 1.1–10.4; *p* = 0.037; Fig. 2). More detailed analysis of the association between reflux oesophagitis and lesions post-PVI revealed that PVI-associated mediastinal oedemas were significantly more frequently observed in patients with pre-existing reflux oesophagitis compared to non-oesophagitis patients (14/22 (64%) vs. 17/45 (38%); *p* = 0.041; Fig. 3A).

Proton pump inhibitors (PPI) represent the gold standard for acid suppressive medical treatment of erosive oesophageal reflux disease. In our cohort, we could identify 14/71 (19.7%) patients with constant PPI medication prior to PVI. Median age and gender distribution did not significantly differ in patients with prior PPI treatment compared to untreated patients (Supplemental Table 4). The major indication for PPI treatment was preventive acid suppression (n = 8) in ASS, NSAR or steroid treated patients, followed by reflux symptoms in the context of a gastro-esophageal reflux disease (n = 3) as well as history of gastritis (n = 1), ulcer (n = 1) and erosive oesophageal reflux disease (n = 1), respectively. Of note, overall incidental findings were equally found in PPI pre-treated patients compared to patients without prior PPI therapy (12/14 (85.7%) versus 41/57 (71.9), p = 0.494). However, a detailed analysis revealed that PPI pre-treated patients suffered significantly less frequently from reflux oesophagitis, assessed directly prior to PVI by EDG, compared to patients without constant PPI intake prior to PVI (1/14 (7%) vs. 21/57 (37%), *p* = 0.049; Fig. 3B), which further underlines the impact of PPI to effectively treat reflux oesophagitis. Due to these observations and the prior results, we hypothesised that constant PPI-treatment prior to PVI might protect against PVI-associated mediastinal lesions by preventing reflux oesophagitis. Indeed, our data demonstrate that mediastinal oedema was observed significantly less frequently in patients with constant PPI prior to PVI compared to non-PPI treated patients (3/14 (21%) vs. 28/53 (53%), *p* = 0.036, Fig. 3C).

## Discussion

The present study shows that aged and obese AF patients have a distinct risk for incidental but clinically relevant pathologies of the upper GI tract. Furthermore, patients with erosive oesophageal reflux disease are at increased risk for PVI-induced mediastinal oedema. Constant PPI treatment prior to PVI seems to reduce PVI-associated mediastinal lesions. Taken together, our study underlines the need to carefully assess the risk profile of AF patients prior to PVI and to consider pre-interventional PPI treatment to reduce risk for PVI associated mediastinal oedema.

### Incidental findings in AF patients scheduled for PVI

AF patients are at special risk for pathologies of the upper GI tract for several reasons: Firstly, comorbidities that trigger and perpetuate AF like diabetes mellitus, obesity, heart failure, sleep apnoea as well as hypertension^1,10,11^ are directly or indirectly associated with acid related upper GI-tract pathologies. This association can be explained by gastral acid secretion due to overactivation of vagal or hormonal stimulation of the parietal cells and disbalance of the gastric mucous membrane^8^. Secondly, reflux itself has been suggested to facilitate AF in many studies: this association of upper gastrointestinal symptoms and arrhythmias was described for the first time in 1912 by Ludwig Roemheld as „gastrocardiac syndrome “^12^ and more recent studies have demonstrated that gastro-oesophageal reflux disease can increase the risk for AF development up to 39%^13–16^ or could detect an association of gastroesophageal reflux disease with an increased risk for AF and a HR of 1.31 to develop AF within the next 3 year^17^. Taken together, these studies strongly underline that AF patients do not exhibit the same risk factors for pathologies of the GI tract compared to a healthy population but rather represent a distinct cohort with an increased risk for incidental findings of the upper GI tract. Indeed, the proportion of clinically relevant incidental findings was higher amongst the AF patients in our cohort compared to published information on GI pathologies in the normal asymptomatic population without cardiac comorbidities^18^. In particular, overall relevant incidental findings such as reflux oesophagitis, mycosis-associated oesophagitis, erosive gastritis or duodenitis, gastral or duodenal ulcer and suspect mucosal and submucosal lesions were present in almost 75% of AF patients. This is in line with the findings of Knoop et al.^19^, who demonstrated that GI pathologies are frequently found in asymptomatic patients with cardiac disorders. Direct comparison of incidental findings though revealed that occurrence of reflux oesophagitis was distinctly higher in our study as compared to the findings of Knoop et al., who found an occurrence of reflux in 12%, which is comparable to the reflux rates observed for the normal population^18^. Nevertheless, other studies confirm our results of higher incidence of erosive oesophageal reflux disease in the AF population^14,20^. In conclusion, AF patients represent a special cohort with an elevated risk for incidental clinically relevant GI pathologies. Especially erosive or ulcerative acid related disease of the upper GI tract are of special importance as all AF patients need oral anticoagulation even in the absence of risk factors for at least 8 weeks after ablation. Lagi et al.^21^ could already demonstrate that AF patients benefit from preventive treatment of incidental upper GI findings before starting oral anticoagulation with significant reduction of associated complications like upper GI-bleeding. Our data showing a distinct risk for AF patients aged older than 50 years for peptic comorbidities including gastritis and gastric ulcer, is in line with the observation of Lagi et al.^21^ and further hint at a strongly increased risk for upper GI-bleeding within this patient cohort.

### Association between acid-related pathologies and safety outcome of PVI

We have already demonstrated that the freeze protocol has a significant impact on oesophageal and mediastinal lesions with significant reduction when using a TTI-guided protocol^6^. Nevertheless, mediastinal oedema > 10 mm still occur in 25% of patients with a TTI-guided freeze protocol. Up until now, predisposing factors for the development of such mediastinal oedema are still not known. It must be stated that mediastinal oedema can only be regarded as surrogate lesions for the development of the fortunately rare atrio-oesophageal fistulae. The pathophysiology of the formation of atrio-oesophageal fistula has not been fully understood yet. However, the delayed development of fistulae after ablation hint at a crucial role of ischaemic lesions of the oesophagus due to thermal injury of the anterior oesophageal arteries^5^, which are localized in a fat pad between the left atrium and oesophagus. It is worthy of note that mediastinal oedemas typically occur in this anatomic region and might therefore represent precursor lesions of atrio-oesophageal fistula.

An association between atrio-oesophageal distances, which was suggested by some studies^19^, could not be confirmed in our recent study^6^. For the first time, we here demonstrate that reflux oesophagitis is significantly associated with a higher risk for PVI-associated mediastinal lesions as an independent risk. Our data clearly demonstrate that pre-existing reflux oesophagitis has distinct impact on PVI safety profile with an increased risk for mediastinal lesions, which was significantly reduced in patients with constant PPI-treatment prior to PVI. Oesophago-mediastinal lesions are regarded as risk factors for the rare but highly lethal PVI-induced complication of an atrio-oesophageal fistula.

Therefore it is important to continue on improving the safety profile of catheter ablation. To our best knowledge the current study is the first, which prospectively identifies risk factors for mediastinal lesions other than freeze protocols in cryoablation patients.

### Pathophysiology

The reason why erosive oesophageal reflux disease is associated with an increased risk for mediastinal lesions still remains unclear but analogous to the hypotheses of an association between gastro-oesophageal reflux disease and AF, patients with acid related disease are probably more vulnerable to mediastinal lesions after PVI due to an increased vagal activity and oesophageal inflammation in the close vicinity of the pericardial structures^16,22,23^. Tougas et al.^23^, for instance, found an association of acid-related irritation of the oesophagus and increasing vagal activity. Reflux of acid can induce localized oesophageal inflammation leading to a changed autonomic innervation with increased vagal nerve stimulation^23^. Due to the close vicinity of the oesophagus and the left atrium, oesophageal injury or irritation can further impact vagal nerve activity as suggested by Linz et al.^16^. Additionally, Chauhan et al.^24^ could demonstrate that oesophageal acid irritation was associated with a reduced coronary blood flow in coronary artery disease patients. As this was not detected in patients after heart transplantation, which includes complete heart denervation, these data hint at cardio-oesophageal reflexes in AF associated with gastro-oesophageal reflux disease^16^ and may be the underlying pathomechanism for acid-related mediastinal oedema after PVI. Indeed, Reddy et al.^25^ demonstrated that vagal responses during radiofrequency ablation occurred significantly more frequently in gastro-oesophageal reflux disease patients as compared to the “non-GI” AF cohort^25^, which further underlines our hypothesis of reflux-associated vagal activation and mucosal inflammation in the vicinity of the LA as an underlying mechanism of mediastinal lesion after PVI.

### Clinical implications and future perspectives

Analogous to the study of Knoop et al.^19^, the investigated AF-PVI patient cohort revealed no manifest symptoms suspicious for upper GI disease like abdominal pain, diarrhoea, emesis, retrosternal pain or dysphagia and highly symptomatic patients were excluded in both studies. This indicates that in non-selected “normal” AF patients the incidence of acid-related disease might be even higher and probably underestimated in the current studies. Our results should increase the awareness for upper GI pathologies in patients suffering from AF. The performance of preventive EGD prior to PVI may be desirable, but it is unlikely that this can be implemented into daily clinical practice without larger randomized trials. To support the results of this retrospective analysis, the routine performance of EGD before AF ablation should be prospectively investigated. Alternatively, prophylactic PPI-treatment started at an adequate time interval before PVI should be discussed. Current studies could demonstrate a correlation between oesophageal pH and AF burden^15,26^ and a significant association between PPI-treatment of reflux disease in AF patients with either reduction or even termination of AF episodes^27^. It is tempting to interpret these observations as a surrogate parameter for termination of acid-related underlying pathomechanism triggering AF by PPI, which would be in line with our findings of reduced PVI-associated mediastinal oedema in patients with constant prior PPI treatment.

## Conclusion

In the present study, we have demonstrated that AF patients have a high risk for clinical relevant findings within the upper GI tract, including acid related diseases like reflux oesophagitis, gastritis and gastric ulcer, which increases in older or obese patients. Furthermore, our data indicate that reflux oesophagitis is associated with a higher risk for development of mediastinal lesions after PVI. Our data therefore lead to the following conclusions: Firstly, even AF patients with no history of gastrointestinal symptoms may benefit from preventive endoscopy and treatment of incidental findings before starting oral anticoagulation. Secondly, AF patients scheduled for PVI may benefit from preventive pre-interventional PPI-treatment to protect from potential acid related inflammation of the upper gastrointestinal tract to reduce PVI-associated mediastinal lesions and bleeding complications due to oral anticoagulations. However, larger randomized-controlled trials are necessary to proof this concept before routinely implementing this preventive pre-interventional PPI-therapy into clinical practice. These trials need to evaluate the efficacy of pre-interventional PPI-treatment to prevent mediastinal lesions as well as the safety and potential side-effects of this additional drug therapy.

## Supplementary Information

Supplementary information.
