# Supplementary material for: Pre-procedural proton pump inhibition is associated with fewer peri-oesophageal lesions after cryoballoon pulmonary vein isolation
Source: Sci Rep. 2021 Feb 25;11:4728. doi: 10.1038/s41598-021-83928-0 (PMC7907235; doi:10.1038/s41598-021-83928-0)
Supplement: Supplementary file 1 — Supplementary information. [file 41598_2021_83928_MOESM1_ESM.docx]

**Pre-procedural proton pump inhibition is associated with fewer peri-oesophageal lesions after cryoballoon pulmonary vein isolation**

**F. Cordes^1,#^, C. Ellermann^2,#,*^, D. G. Dechering^2^, G. Frommeyer^2^, S. Kochhäuser^2^, P. S. Lange^2^, C. Pott^3^, F. Lenze^1^, I. Kabar^1^, H. Schmidt^1^, H. Ullerich^1,#^, L. Eckardt^2,#^**

^1^ Department of Medicine B, Gastroenterology and Hepatology, University Hospital

Muenster, Muenster, Germany

^2^ Department of Cardiology II (Electrophysiology), University Hospital Muenster,

Muenster, Germany

^3^ Department of Cardiology, Schuechtermann-Klinik, Bad Rothenfelde, Germany

# The first and last two authors contributed equally to the study.

**Address for correspondence:**

Dr. med. Christian Ellermann

Klinik für Kardiologie II ¬ Rhythmologie

Universitätsklinikum Münster

Albert-Schweitzer Campus 1

D-48149 Münster

Phone: (+49) 251-834 4932

Fax: (+49) 251-834 9965

Email: christian.ellermann@ukmuenster.de

SUPPLEMENTAL DIGITAL CONTENT

SUPPLEMENTAL TABLES

| **Supplemental Table 1:** Demographics and comorbidities of the MADE-PVI cohort^1^ | **Patients** |  |  |
| --- | --- | --- | --- |
| **Demographics**  Age at PVI, yrs [median, IQR]  Patients / male [n]  Body mass index [mean (SD)] | 60 (16)  71/53  27.4 (3.6) |  |  |
| **Comorbidities**  Hypertension [n (%)]  Diabetes mellitus [n (%)]  Ischemic cardiomyopathy [n (%)]  Non-ischemic cardiomyopathy [n (%)] | 37 (52)  5 (7)  5 (7)  6 (8.5) |  |  |

BMI, body-mass-index; IQR, inter-quartile-range; n, number; PVI, pulmonary vein isolation; SD,

standard deviation

**Reference:**

1 Cordes, F. *et al.* Time-to-isolation-guided cryoballoon ablation reduces oesophageal and mediastinal alterations detected by endoscopic ultrasound: results of the MADE-PVI trial. *Europace* **21**, 1325-1333 (2019)

**Supplemental Table 2:** Demographics and incidental findings of patients aged older versus younger than 50 years.

|  | **Patient cohort ≤50 years** | **Patient cohort > 50 years** | ***P-value*** |
| --- | --- | --- | --- |
| **Demographics**  Age at PVI, yrs [median, IQR]  Patients / male [n]  Body mass index [mean (SD)]  **Incidental findings**  Erosive oesophageal reflux disease [n (%)] | 46.5 (9)  14/13  26.8 (4.1)  6 (42.9) | 62 (11.5)  57/40  27.5 (3.5)  16 (28.1) | 0.098  0.543  0.224 |
| Barrett´s oesophagus [n (%)]  Mycosal oesophagitis [n (%)]  Hiatal hernia [n (%)] | 1 (7.1)  1 (7.1)  **1 (7.1)** | 13 (22.8)  3 (5.3)  **19 (33.3)** | 0.187  0.593  **0.045** |
| Moderate / severe gastritis [n (%)]  Deep erosion / ulcer [n (%)]  Polypoid lesions [n (%)]  Malignoma [n (%)] | **3 (21.4)**  0 (0)  1 (7.1)  0 (0) | **32 (56.1)**  9 (15.8)  5 (8.8)  1 (1.8) | **0.020**  0.112  0.663  1.000 |

BMI, body-mass-index; IQR, inter-quartile-range; n, number; PVI, pulmonary vein isolation; SD, standard deviation

| **Supplemental Table 3:** Demographics and incidental findings of obese versus non-obese patients | **Patient cohort BMI < 30 kg/m^2^** | **Patient cohort BMI ≥ 30 kg/m^2^** | ***P-value*** |
| --- | --- | --- | --- |
| **Demographics**  Age at PVI, yrs [median, IQR]  Patients / male [n]  Body mass index [mean (SD)]  **Incidental findings**  Moderate to severe erosive reflux disease [n (%)] | 60.0 (16.25)  47/34  25.2 (2.1)  **1 (2.2)** | 60.5 (15.75)  24/19  31.4 (1.9)  **4 (16.7)** | 0.937  0.580  <0.001  **0.044** |
| Barrett´s oesophagus [n (%)]  Mycosal oesophagitis [n (%)]  Hiatal hernia [n (%)] | 9 (19.2)  3 (6.5)  13 (28.3) | 5 (20.8)  1 (4.2)  7 (29.2) | 0.597  0.575  0.574 |
| Moderate / severe gastritis [n (%)]  Deep erosion / ulcus [n (%)]  Polypoid lesions [n (%)]  Malignoma [n (%)] | 21 (45.7)  5 (10.9)  2 (4.3)  0 (0) | 14 (48.3)  4 (16.7)  4 (16.7)  1 (4.2) | 0.225  0.368  0.100  1.000 |

BMI, body-mass-index; IQR, inter-quartile-range; n, number; PVI, pulmonary vein isolation; SD, standard deviation

**Supplemental Table 4:** Demographics and characteristics of PPI pre-treated patients versus patients without PPI pre-treatment.

|  | **PPI pre-treatment** | **No PPI pre-treatment** | ***P-value*** |
| --- | --- | --- | --- |
| **Demographics**  Age at PVI, yrs [median, IQR]  Patients / male [n]  **Acid-related incidental findings**  Erosive oesophageal reflux disease [n (%)] | 60.0 (16)  14/8  1 (7.1) | 60.0 (16)  57/45  21 (36.8) | **0.049** |
| Barrett´s oesophagus [n (%)] | 3 (21.4) | 11 (19.3) | 0.730 |
| Moderate/ severe gastritis [n (%)]  Ulcer [n (%)] | 10 (71.4)  0 (0) | 25 (43.9)  5 (8.8) | 0.079  0.677 |
| **Indication for PPI pre-treatment**  Gastro-esophageal reflux disease [n (%)]  Erosive oesophageal reflux disease [n (%)]  Gastritis / ulcer [n (%)]  Prophylactic therapy [n (%)] | 3 (21.4)  1 (7.1)  2 (14.3)  8 (57.1) | **-**  **-**  **-**  **-** |  |

IQR, inter-quartile-range; n, number; PVI, pulmonary vein isolation; PPI, protone pump inhibition; SD, standard deviation

SUPPLEMENTAL FIGURES

**Supplemental Figure 1: Incidental findings in obese patients with a BMI > 30 kg/m^2^.** (**A**) Clinical relevant incidental findings did not occur significantly different in obese (**BMI > 30 kg/m^2^**) versus non-obese (**BMI < 30 kg/m^2^**) patients (*p*=0.259). (**B**) However, moderate to severe erosive oesophageal reflux disease (LA classification B-D^6^) was detected significantly more often in obese versus non-obese patients (*p*=0.044). Shown is the number of patients with clinical relevant incidental findings (**A**) and erosive oesophageal reflux disease (**B**).

**A**

***p*=0.259**

No obesity (BMI < 30 kg/m^2^)


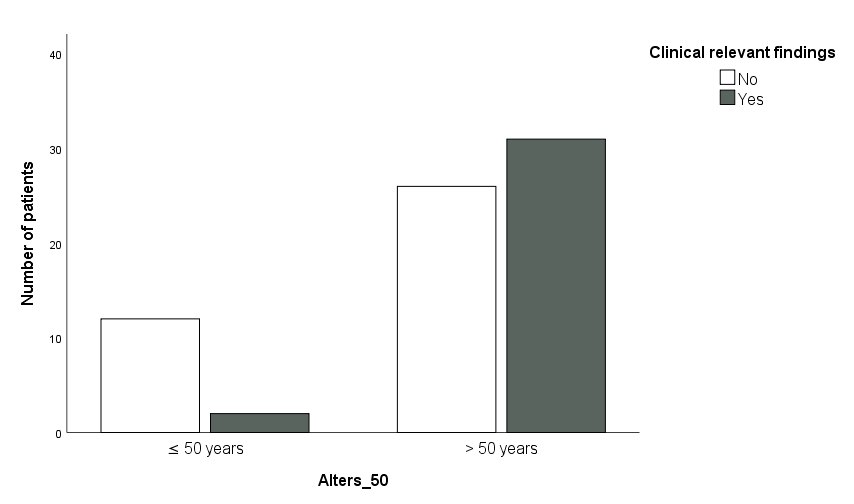


No relevant finding

Relevant findings

Number of patients

0

10

20

30

Obesity (BMI ≥ 30 kg/m^2^)


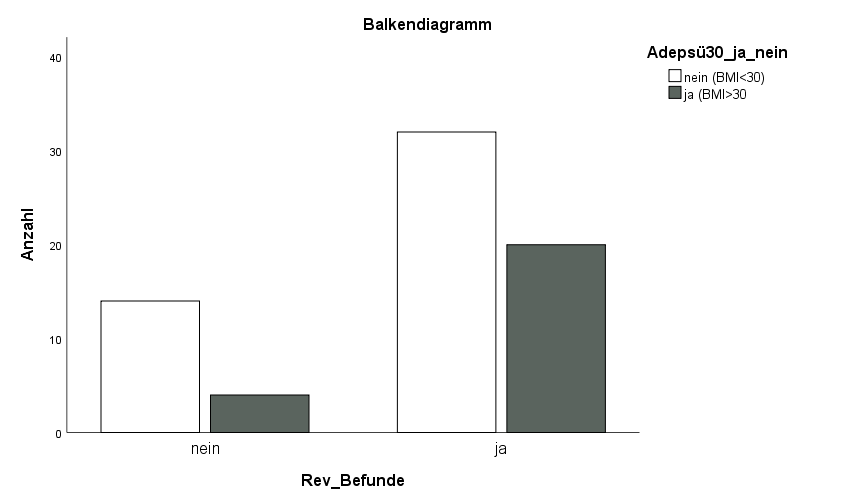


40


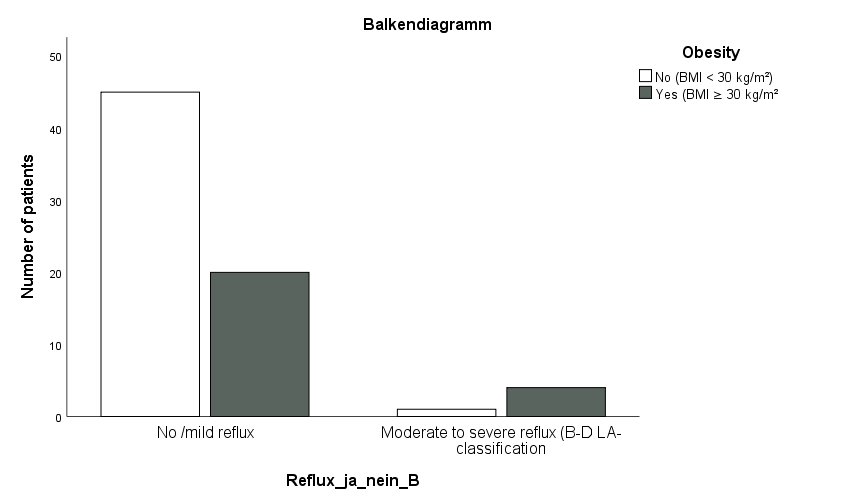


**B**

***p*=0.044**

No obesity (BMI < 30 kg/m^2^)


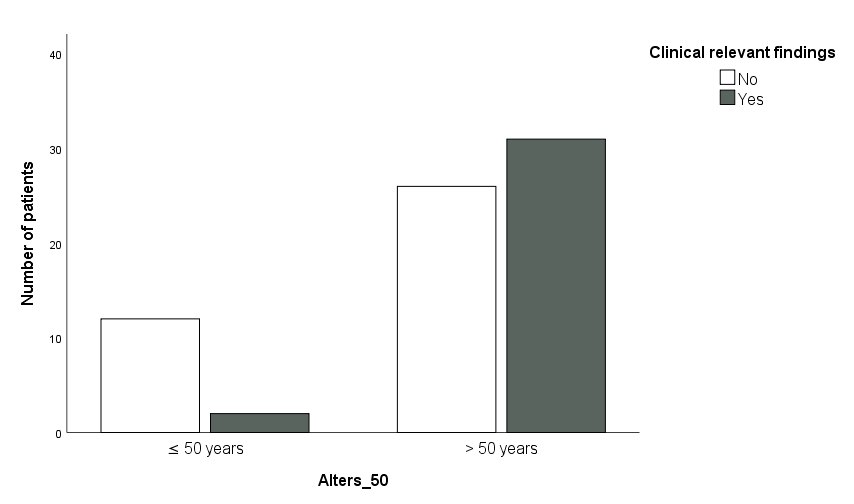


No / mild erosive reflux

Moderate to severe erosive reflux

Number of patients

0

10

20

30

Obesity (BMI ≥ 30 kg/m^2^)

40

50
